# Special Issue “Feature Papers in Population and Evolutionary Genetics and Genomics”

**DOI:** 10.3390/genes14010038

**Published:** 2022-12-23

**Authors:** Maria-Anna Kyrgiafini, Zissis Mamuris

**Affiliations:** 1 Laboratory of Genetics, Comparative and Evolutionary Biology, Department of Biochemistry and Biotechnology, University of Thessaly, Viopolis, Mezourlo, 41500 Larissa, Greece; 2 Averofeio Agri-Food Technological Park of Thessaly, University of Thessaly, Gaiopolis, 41336 Larissa, Greece

Theodosius Dobzhansky famously wrote in 1973 that “nothing in biology makes sense except in the light of evolution” [1], laying the foundations for the Modern Synthesis that combined population genetics with evolutionary biology and bridged the gap between geneticists and naturalists. Population genetics study the genetic diversity and structure of populations and all the processes involved [2]. Though the term Modern Synthesis is referred to in Huxley’s book, *Evolution: The Modern Synthesis*, published in 1942 [3], many years passed before population genetics was established as an integral part of evolutionary biology. Many years later, the gap that exists between macroevolutionary thinking and the microevolution of local populations is also reflected in Joe Felsenstein’s words: “systematists and evolutionary geneticists do not often talk to each other” [4]. However, between then and now, approximately thirty years after this quote, astonishing changes and progress have been made. The development of next-generation sequencing (NGS) technology has enabled the production of genomic data for huge population samples, as well as the rapid increase in computational power and simulation modeling, allowing scientists to study complex evolutionary processes. Today, population genetics theory is inarguably the theoretical backbone of the Modern Synthesis, and all this progress and the combination of population and evolutionary genetics and genomics have provided answers to biologically important questions in a wide range of areas of research.

This Special Issue, “Feature Papers in Population and Evolutionary Genetics and Genomics”, includes nine fascinating and novel articles that address a wide range of important topics relevant to evolutionary and population genetics. The papers encompass human and non-human studies, and they include both original research articles and review articles.

Firstly, the explosion of human genetic data in the past two decades has increased our understanding of genetic diversity among human populations. Genetic diversity is an exciting field, as these genomic regions have a story to tell about population admixture, the origins of populations, migration, adaptation to different environments, and human evolution and history, in general. Fedorova, Khrunin et al. (2022) [5] analyzed common single-nucleotide polymorphisms (SNPs) and identified distinct genomic patterns among human populations, shedding light on worldwide genetic diversity. They studied eighty-four thousand abundant region-specific alleles (ARSA) that are common in one continent but not in the rest of the world and approximately seventeen thousand polymorphic sites with a regional absence of common alleles (RACAs) that are widespread globally but absent in one continent. They identified unique combinations of RACAs and ARSAs in every geographic region, and they analyzed the genes on which the RACA and ARSA SNPs were mapped, observing that the largest portion of genes shared by multiple populations are associated with the nervous system. All these ARSA and RACA SNPs would also be valuable candidates for studies exploring population structures or evolution, especially genome-wide association studies (GWAS), since these polymorphic sites are rich in functional alleles with a variety of adaptive roles. The study of Urnikyte, Molyte, and Kučinskas (2021) [6] also examined genetic diversity, focusing on a specific Baltic population. Using a genome-wide approach, they unrolled the history of the Lithuanian population. Among other findings that add to our understanding of microevolutionary processes involving specific human populations, they reported that Lithuanians are genetically close to their neighboring populations (Latvians, Estonians, Belarusians) and, to a lesser extent, to West and South Slavs. Furthermore, the authors estimated the times of divergence between Lithuanians and other populations and identified regions under positive selection that have not been reported in the past.

However, the methodological approach and interpretation of the results are two very important issues that should be considered with caution and in detail in all evolutionary and population genetic studies. In line with this, Subramanian (2022) [7] conducted a very interesting study about the fixation index (FST), a tool widely used for the measurement of population differentiation due to genetic structure, and added scientific knowledge to the discussion about the impacts of conservation of genes in regard to FST measurement. Although this is not the first time that a reduction in the FST estimates of selectively constrained sites has been reported in the literature, this study provides an answer to the question of why this is and has been observed. More specifically, it is suggested that the reduction in FST observed at the constraint sites is caused by an excess in the fraction of deleterious variants causing segregation within populations compared to those causing segregation between populations. Given the importance of FST, which is used in many studies ranging from evolutionary and ecological studies to clinical genetic studies, these findings are of scientific importance and could contribute to both better interpretation and the design of future studies. Maceda and Lao (2022) [8] also questioned how the robustness of the 1000 Genomes (1000 G) Project database is affected by the sequencing centers involved in the project, leading to potential biases. This is arguably an important question to address, as the 1000 G datasets are used very often in research ranging from medical genetics and genetic epidemiology studies to evolutionary studies. Therefore, the authors showed that the data produced by different centers are not homogeneous and that differentiation is greater among rare variants with a minor allele frequency (MAF) < 0.2%. This should also be considered when interpreting the results of population genetic studies, and in addition, the batch effect described by the authors could lead to better experimental designs of relevant studies. Finally, ultra-conserved elements (UCEs) are among the most widely used DNA markers in phylogenomic and evolutionary studies. These are DNA regions with a length > 200 bp that remain unchanged for hundreds of millions of years in vertebrates, as they are shared between humans, mice, and rats [9]. Despite their broad use and the fact that many years have passed since their discovery, their function remains a mystery. Fedorova et al. (2022) [10] performed a bioinformatics analysis and observed that the GpC dinucleotide is more abundant than expected within ultra-conserved noncoding elements (UCNEs), and as this dinucleotide has a lower dissociation rate than any other combination of nucleotides, they suggested that this increased stability might contribute to a unique 3D conformation of the DNA molecule, even favoring the specificity of binding proteins. This statement requires further experimental validation, but it offers a new perspective on the mystery of UCNEs, and it could provide valuable information for studies utilizing them as markers.

Of equally special interest are the papers included in this Special Issue investigating non-model organisms, their genetic diversity, evolution, and adaptation to unstable and extreme habitats. Castel et al. (2022) [11] explored the evolution of life in hydrothermal vents that are created and become extinct according to the ongoing movement of the Earth’s tectonic plates. These events, in combination with the extreme thermal and chemical conditions, create complex ecosystems that are intriguing for the study of adaptation and speciation events. Therefore, Castel et al. (2022) [11] studied three species of the genus *Alviniconcha* (Gastropoda: Abyssochrysoidea) that inhabit active Western Pacific vent fields. Based on their analysis, the authors presented the distribution of every species and reported that the three species are genetically divergent and phenotypically different. However, they also reported a surprising result, as they indicated gene flow during secondary contacts, despite the divergence of the species (reflected on both nuclear and mitochondrial genomes), following a long isolation period. The authors offered some suggestions about the barriers responsible for the maintenance of the distinct species and the divergence observed but highlighted that these should be further investigated. Furthermore, Lim, Habib, and Chen (2021) [12] conducted a comparative phylogenetic and phylogenetic study of pennah croakers, a demersal fish species, in Southeast Asian (SEA) waters, where the highest species diversity worldwide is observed. The researchers investigated the phylogeny, genetic diversity, biogeography, and distribution of pennah croaker species and populations. They concluded that climatic and oceanographic alterations, such as cyclical glaciations, which created physical barriers, as well as species habitat preferences, could explain the phylogeographic patterns and the distribution of the species studied, potentially also explaining the biodiversity observed in SEA waters. As the reasons behind this extreme biodiversity of marine species are not yet fully clarified, this study is of significance and adds to our knowledge about biogeography and the evolution of marine organisms.

Finally, organelle genomes can also provide a better understanding of evolution, speciation, and population divergence, and mitochondrial DNA (mtDNA), with its intrinsic properties, is extremely valuable for evolutionary and population genetic studies. Parakatselaki et al. (2022) [13] reported a case of insertion of a large nuclear mitochondrial fragment (NUMT) into the X chromosome of *Drosophila melanogaster*. Surprisingly, the size of this NUMT was also almost equal to the size of the whole mtDNA. NUMTs were discovered approximately two decades ago, and they are often characterized as mitochondrial pseudogenes, since they are copies of the mitochondrial genome located on the nuclear genome [14]. As Parakatselaki et al. (2022) [13] highlighted, these NUMTs can lead to false results and cause confusion about the biparental inheritance of mtDNA. These findings raise intriguing questions and are of importance for the debate about the biparental inheritance of mtDNA in humans [15]. It is also made clear that before drawing conclusions about the biparental inheritance of mtDNA, it is very important to verify and exclude the possibility of NUMT presence. Kyrgiafini et al. (2022) [16] reviewed a similarly intriguing phenomenon associated with the maternal inheritance of mtDNA and mito-nuclear incompatibility, the phenomenon of the Mother’s Curse, or the bad luck of being male, as the phenomenon could also be described, because mutations that are harmful to males tend to accumulate more easily in mtDNA. The authors summarized the studies on the association of the Mother’s Curse with diseases that affect male fitness and reported cases of the Mother’s Curse that lead to hybrid breakdown in both nature and the laboratory due to mito-nuclear incompatibility. All these findings are extremely important within the scope of the development of novel approaches, such as the three-parent technique, which makes use of the mtDNA of a third parent. Moreover, as Kyrgiafini et al. (2022) [16] highlighted, the Mother’s Curse may also hold promise for potential applications aiming to address global challenges, such as pest management, by using male-harmful mutations that affect reproductive capacity, enabling the population suppression of pests.

In conclusion, the articles included in this Special Issue cover a wide range of topics, including human population studies, studies of organisms inhabiting extreme environments, and studies about mtDNA, and prove that the combination of population and evolutionary genetics can provide answers to many biological questions. We anticipate that this collection of articles will set the stage for exciting future investigations in the field of evolutionary and population genetics and genomics.

## Data Availability

Not applicable.
